# Supplementary material for: Can Public Spaces Effectively Be Used as Cleaner Indoor Air Shelters during Extreme Smoke Events?
Source: Int J Environ Res Public Health. 2021 Apr 13;18(8):4085. doi: 10.3390/ijerph18084085 (PMC8070163; doi:10.3390/ijerph18084085)
Supplement: Supplementary file 1 [file ijerph-18-04085-s001.pdf]

Supplementary Materials: Checklist to assess the library as a cleaner indoor air shelter

Table S1. NCPHU Community Cleaner Air Shelter Practical Assessment Checklist.

Aim: to identify a building available during daytime hours for community members to freely utilize for cleaner air respite (no animals).

**Common Name of location**	
**Address**	
**Building owner**	
**Building occupier**	
**Facility Manager/HVAC contractor**	

**Criteria**	**Description**	**0 none**<br>**1 ok**<br>**2 good**
Opening hours (modification options?)		
Parking		
Bus		
Disabled access		
HVAC Brand & model		
Date of commissioning/installation		
Filter nature & type<br>o Mechanical filter-based<br>(flat/pleated HEPA: particle size?)<br>o Electrostatic precipitating<br>o Ion generators<br>o Carbon filters or ozone generators		
Air exchange rate – variability		
Location of air ducts into room/s		
Ability to close inlets at night		
HVAC Manual/User guide		
R&M schedule available		
R&M records available		
Last service/filter change		
Plan of HVAC system		
Odour indoors at time of assessment		
# Chairs / seats / couches for use		
# toilets male & female & disabled		

Drinking water availability		
Food availability		
Rubbish bins availability		
Telephone availability		
Max # persons rated		
Space for comm. engagement officer		
Other comment		

EHO

Name____Date________Time_

____

Supplementary Material: Side by Side Comparison of Two Smog Units at Each of the Library Sites

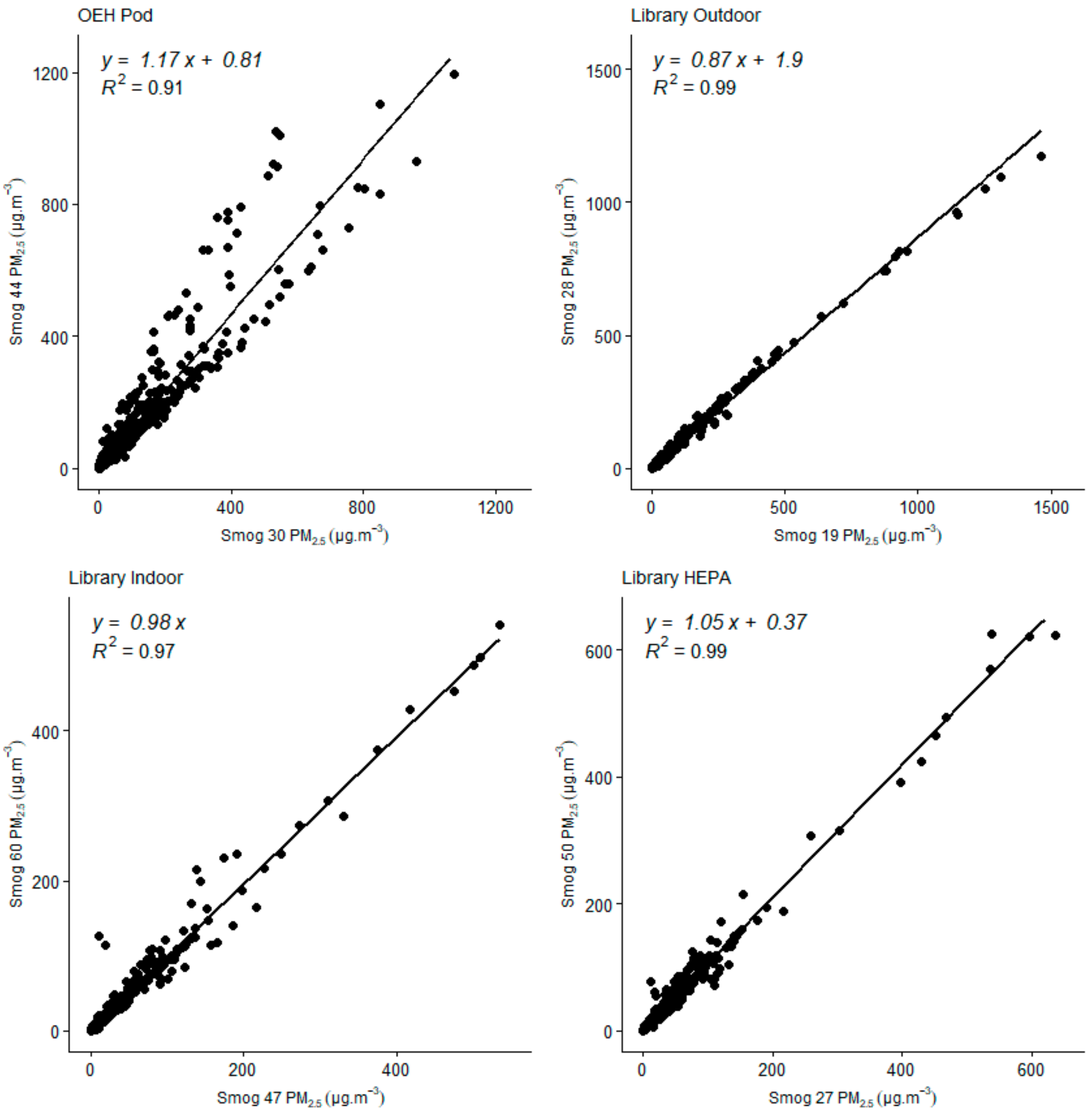

Figure S1. Port Macquarie study – SMOG unit pair correlations.
